# Collision Tumor of Angioimmunoblastic T-Cell Lymphoma and Kaposi Sarcoma in an HIV-Negative Elderly Woman: The First Reported Case in Asia

**DOI:** 10.3390/diagnostics15182411

**Published:** 2025-09-22

**Authors:** Myung-Won Lee, Jin-Man Kim

**Affiliations:** 1 Department of Internal Medicine, Chungnam National University Hospital, 282 Munhwa-ro, Jung-gu, Daejeon 35015, Republic of Korea; iyoo23@naver.com; 2 Department of Pathology, Chungnam National University College of Medicine, 266 Munwha-ro, Jung-gu, Daejeon 35015, Republic of Korea

**Keywords:** angioimmunoblastic T-cell lymphoma, Kaposi sarcoma, collision tumor, HIV-negative, HHV-8, lymphadenopathy

## Abstract

**Background/Objectives:** Angioimmunoblastic T-cell lymphoma (AITL) is a rare peripheral T-cell lymphoma of follicular helper T-cell (TFH) origin, often associated with immune dysregulation and EBV-positive B-cell proliferation. Kaposi sarcoma (KS) is a vascular neoplasm caused by human herpesvirus 8 (HHV-8), typically arising in immunocompromised individuals. The synchronous occurrence of AITL and KS in HIV-negative patients is exceptionally rare, with only three cases previously reported worldwide. **Case Presentation:** We describe an 81-year-old HIV-negative Korean woman presenting with progressive generalized edema and dyspnea. Imaging revealed multifocal lymphadenopathy. Excisional biopsy of the inguinal lymph node showed two distinct but adjacent neoplastic processes. The AITL component demonstrated a polymorphous infiltrate of atypical TFH cells expressing CD3, CD4, CD10, PD-1, and Bcl-6, with monoclonal TCR-γ rearrangement and TET2 and RHOA mutations. The KS component comprised spindle cells with slit-like vascular spaces, red blood cell extravasation, and immunoreactivity for HHV-8, CD31, CD34, and ERG. The findings were consistent with a collision tumor. Despite supportive care, the patient’s condition deteriorated, and she was discharged with palliative care. **Discussion:** The coexistence of AITL and KS in an HIV-negative setting raises important pathogenetic considerations. AITL is characterized by profound immune dysregulation, with depletion of normal T-cell subsets, abnormal B-cell activation, and cytokine milieu changes that may favor latent viral reactivation. This immunologic environment may permit HHV-8 reactivation, thereby facilitating the development of KS even in the absence of overt immunodeficiency due to HIV infection. Our findings support the hypothesis that AITL-related immune dysfunction may create a permissive niche for HHV-8-driven neoplasia. **Conclusions:** This is the first reported case in Asia and the fourth worldwide of a collision tumor comprising AITL and KS in an HIV-negative patI dient. The case suggests that AITL-associated immune dysregulation may facilitate HHV-8 reactivation and KS development even in the absence of HIV infection. Awareness of this association is critical for accurate diagnosis and optimal patient management.

## 1. Introduction

Angioimmunoblastic T-cell lymphoma (AITL) is a mature T-cell tumor classified under peripheral T-cell lymphomas (PTCLs), specifically derived from CD4+ follicular helper T (TFH) cells [1,2,3]. It is associated with dysregulated immune responses, systemic constitutional symptoms, generalized lymphadenopathy and frequently with Epstein–Barr virus (EBV)–positive non-neoplastic immunoblastic B-cells. Pathologic findings show polymorphous infiltrate of neoplastic T cells with clear cytoplasm and small to medium-sized irregular nuclei admixed with B-immunoblasts, plasma cells and eosinophils. It shows prominent arborizing high endothelial venules (HEVs) and expanded follicular dendritic cell (FDC) meshworks. The immunophenotype reveals positive for T-cell markers (CD2, CD3, CD4) and TFH markers (CD10, Bcl-6, PD-1, CXCL13, ICOS) [4,5,6,7]. Molecular features of TET2, DNMT3A, RHOA mutation are frequently found [8,9,10].

Kaposi sarcoma (KS) is a vascular tumor associated with infection by human herpesvirus 8 (HHV-8) and is characterized by spindle cell proliferation, slit-like vascular spaces, and inflammatory infiltrates [11,12]. It commonly affects the skin but may involve lymph nodes and visceral organs. KS has four epidemiologic and clinical subtypes, all associated with HHV-8 infection. These include the classic, endemic (African), epidemic (AIDS-related), and iatrogenic (Post-transplant) forms [13]. The neoplastic spindle cells express CD31, CD34, ERG, and HHV-8 LANA-1.

A collision tumor comprising AITL and KS in an HIV-negative patient is exceedingly rare. Only three nodal cases have been documented globally, none from Asia [14]. This report describes the first known Asian case.

## 2. Case Presentation

**Figure 1:**
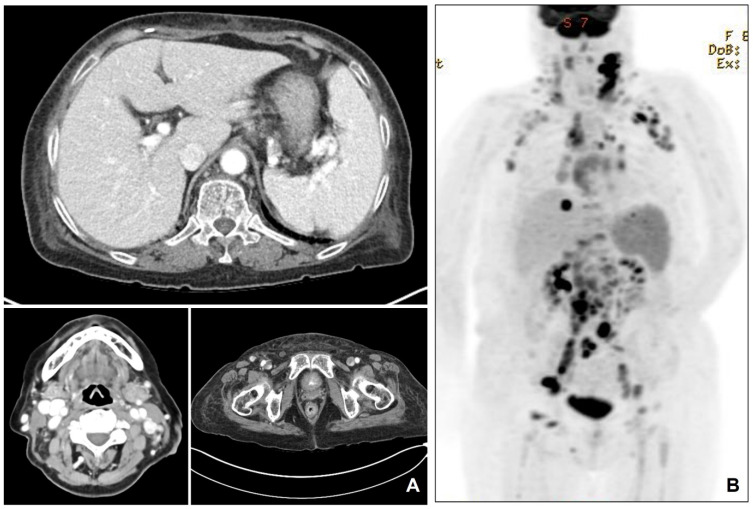
The CT scan shows splenomegaly, and multifocal hypervascular lymphadenopathy, including neck, abdomen, and inguinal area (**A**). The PET-CT shows multifocal involvement of lymph nodes, spleen and bone marrow (**B**).

An 81-year-old woman presented with a 3-month history of progressive generalized edema and worsening dyspnea. Physical examinations showed bilateral lower extremity pitting edema and palpable lymphadenopathy in the cervical, axillary, inguinal, and abdominal regions. Laboratory investigations demonstrated mild anemia, elevated lactate dehydrogenase, and negative HIV serology. Whole-body computed tomography (CT) scans performed upon admission showed diffuse lymphadenopathy affecting the cervical, mediastinal, axillary, abdominal, and inguinal regions, as well as splenomegaly (Figure 1).

**Figure 2:**
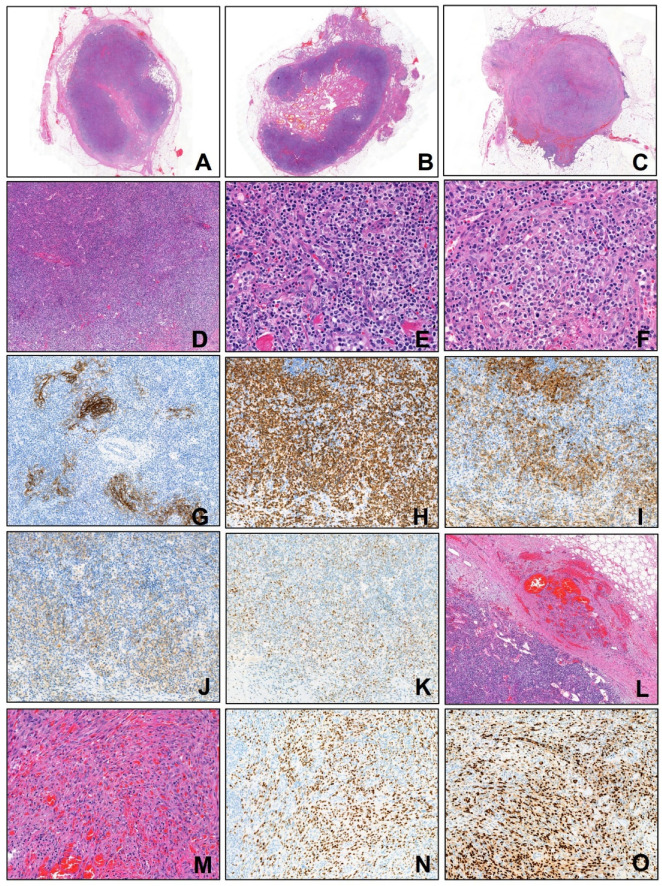
(**A**–**C**) Scan view images of three lymph nodes show coexistence of Angioimmunoblastic T-cell lymphoma (AILT) and Kaposi sarcoma (KS). (**D**–**K**) AILT areas show diffuse effacement of lymph node architecture (**D**), polymorphous lymphoid cell infiltration with high endothelial venules (**E**), atypical lymphoid cells with clear cytoplasm (**F**). CD21 staining shows extrafollicular follicular dendritic cells meshworks (**G**). Immunohistochemistry shows the neoplastic clear cells are positive for CD3 (**H**), CD10 (**I**), PD-1 (**J**), and Bcl-6 (**K**). (**L**–**O**) KS areas show capsular and pericapsular spindle cell proliferation with red blood cell extravasation and slit-like vascular spaces (**L**,**M**). The neoplastic spindle tumor cells express HHV-8 LANA-1 (**N**) and ERG (**O**).

An excisional biopsy of inguinal lymph nodes was performed. Formalin-fixed, paraffin-embedded (FFPE) tissue sections were subjected to immunohistochemical staining using the Dako Omnis automated immunostainer (Dako, Agilent Technologies, CA, USA) according to the manufacturer’s standardized protocols. After deparaffinization, rehydration, and appropriate antigen retrieval procedures, the sections were incubated with the following primary antibodies: CD3 (Dako, 1:300), CD4 (Dako, 1:200), CD10 (Novocastra, 1:100), CD20 (Dako, 1:200), CD21 (Novocastra, 1:100), BCL6 (Dako, 1:50), CD31 (Dako, 1:300), CD34 (Dako, 1:300), anti-ERG (Roche, prediluted), HHV-8 (Cell Marque, prediluted), and PD-1 (Cell Marque, 1:500). The immunoreactions were developed using 3,3′-diaminobenzidine (DAB) as the chromogenic substrate, followed by counterstaining with hematoxylin. Slides were subsequently dehydrated, cleared, and coverslipped for microscopic evaluation. Histopathological examination showed two distinct but intermingled neoplastic components: one component displayed effaced nodal architecture with atypical clear cells positive for CD3, CD4, CD10, PD-1, and Bcl-6, consistent with AITL; the second component demonstrated red blood cell extravasation, spindle cell proliferation forming slit-like vascular spaces, positive for HHV-8 LANA-1, ERG, CD31 and CD34, consistent with Kaposi sarcoma (Figure 2). There was no evidence of cutaneous KS lesions. TCR-gamma gene rearrangement study demonstrated monoclonality. High-throughput sequencing identified TET2 and RHOA (G17V) mutations, which are characteristic of AITL. A positron emission tomography-computed tomography (PET-CT) scan demonstrated extensive involvement of the lymph nodes, spleen, and bones (Figure 1). Treatment with weekly vinblastine (3 mg/m^2^) was initiated. Despite receiving a second dose of vinblastine, the patient’s generalized edema worsened, and her shortness of breath continued without improvement. Supportive care including diuretics and corticosteroids was ineffective. Considering the patient’s advanced age and overall clinical condition, the medical team, along with the patient and her family, agreed not to pursue further aggressive treatments, such as mechanical ventilation. As a result, the patient was discharged home with terminal care measures. Written informed consent for publication of this case report was obtained from the patient. Approval for this case report was obtained from the Institutional Review Board (IRB) of Chungnam National University Hospital (CNUH-IRB 2025-08-002).

## 3. Discussion

The synchronous occurrence of angioimmunoblastic T-cell lymphoma (AITL) and Kaposi sarcoma (KS) in a single patient represents an extraordinarily rare pathological entity. While both diseases are well recognized individually—AITL as a peripheral T-cell lymphoma derived from follicular helper T-cells [1], and KS as a vascular neoplasm caused by human herpesvirus-8 (HHV-8) [11]—their co-existence in the same lymph node and in an HIV-negative individual poses unique challenges in pathogenesis, diagnosis, and clinical management. Only a handful of such cases have ever been reported worldwide [14], making this presentation not only the first documented case in Asia but also an important contribution to the understanding of how immune dysregulation and viral oncogenesis may intersect in lymphoid tissues.

Collision tumors are defined as two histogenetically distinct neoplasms occurring simultaneously within the same anatomical site without histologic admixture [14]. The distinction between true collision tumors and composite tumors is important: in the latter, divergent differentiation arises from a common progenitor cell, whereas in the former, two separate malignancies arise independently but coexist in close proximity. The synchronous occurrence of a T-cell lymphoma such as AITL with a vascular neoplasm like KS is virtually unprecedented and underscores the profound immunologic disturbances associated with AITL. This case demonstrates that the immune microenvironment created by AITL can be permissive for the development of a second, unrelated neoplasm, in this instance HHV-8–driven KS. The rarity of this phenomenon raises important diagnostic and conceptual questions. For the practicing pathologist, the recognition that more than one neoplastic process can exist in the same biopsy specimen is critical. The polymorphous infiltrate of AITL may obscure the vascular spindle cell proliferation of KS, especially if the latter is subtle. Conversely, exuberant vascular proliferation associated with KS may be mistaken for the proliferation of high endothelial venules that are a hallmark of AITL. Careful histomorphologic assessment, judicious use of immunohistochemistry, and correlation with molecular findings are essential for avoiding misdiagnosis.

AITL is recognized as one of the most immune-dysregulating hematologic malignancies [3,15]. Its pathogenesis follows a multistep model in which early mutations in epigenetic regulators such as TET2 and DNMT3A occur in hematopoietic stem cells, producing clonal hematopoiesis [8,16]. Subsequent lineage-specific mutations, particularly the RHOA G17V and IDH2 R172 mutations, occur in follicular helper T-cells, driving their neoplastic transformation [17,18]. These neoplastic TFH cells elaborate a range of cytokines and chemokines, including interleukin-6, interleukin-21, CXCL13, and vascular endothelial growth factor. Such molecules remodel the lymph node microenvironment, stimulate angiogenesis, attract B-cells, and promote abnormal immune responses [3]. Clinically, AITL is notorious for systemic manifestations such as autoimmune cytopenias, polyclonal hypergammaglobulinemia, and susceptibility to infections. Pathologically, it is characterized by effaced lymph node architecture with polymorphous infiltrates, arborizing high endothelial venules, and expanded follicular dendritic cell networks. Immunohistochemistry reveals expression of both pan-T markers and TFH-associated antigens such as PD-1, Bcl-6, CXCL13, and ICOS [1,15]. Importantly, the neoplastic environment of AITL is frequently accompanied by reactive Epstein–Barr virus–positive B-immunoblasts, underscoring the central role of immune dysregulation in this disease. This permissive immune landscape created by AITL provides fertile ground for viral reactivation and secondary neoplasms. Just as EBV-driven B-cell proliferations often accompany AITL, it is plausible that HHV-8 can exploit this dysregulated immune state to drive the development of Kaposi sarcoma even in the absence of HIV infection. Thus, AITL not only represents a malignancy of T-cells but also acts as an orchestrator of secondary oncogenic processes within its disturbed microenvironment.

Kaposi sarcoma is a vascular tumor tightly linked to HHV-8 infection [11,12]. The virus establishes latency in endothelial and B-cells, expressing latent nuclear antigen-1 and a restricted set of viral genes. Under conditions of immune suppression, HHV-8 reactivates and promotes the proliferation of spindle cells, which are considered to be of lymphatic endothelial origin [19]. Viral proteins mimic cellular signaling molecules, activating angiogenic pathways and inhibiting apoptosis [12]. The presence of slit-like vascular spaces, red blood cell extravasation, and characteristic immunoreactivity for CD31, CD34, ERG, and HHV-8 are diagnostic hallmarks.

Traditionally, KS occurs in four epidemiologic settings [11]: the classic indolent form in elderly men of Mediterranean descent, the endemic African form, the epidemic AIDS-related form, and the iatrogenic post-transplant form. In all scenarios, immune dysfunction is central to pathogenesis. What is unusual in this case is that the patient was HIV-negative, elderly, and not immunosuppressed by transplantation, yet developed nodal KS in the setting of AITL. This strongly suggests that the immune disruption intrinsic to AITL is sufficient to permit HHV-8 reactivation and KS tumorigenesis.

The coexistence of AITL and KS highlights the intersection between lymphoid malignancy-driven immune dysregulation and virus-mediated oncogenesis. Several mechanisms may underlie this association [12,20]. First, neoplastic TFH cells in AITL produce high levels of cytokines such as interleukin-6 and vascular endothelial growth factor, which are known to promote angiogenesis and endothelial proliferation, thereby providing a favorable niche for KS development. Second, the immunosuppressive milieu of AITL, characterized by depletion of normal T-cell subsets and impaired immune surveillance, facilitates HHV-8 reactivation. Third, EBV-positive B-cell expansions commonly observed in AITL may further contribute to immune exhaustion, synergizing with HHV-8-driven oncogenesis. Taken together, these factors create a microenvironment in which KS can arise even in patients without classical risk factors such as HIV infection or iatrogenic immunosuppression.

Prior to this report, only three cases of collision tumors comprising AITL and KS had been documented worldwide [14]. These cases shared several clinical features with our patient, including advanced age and absence of HIV infection. However, there were also notable differences in terms of geographic distribution, treatment responses, and outcomes. Some patients presented with cutaneous KS lesions, whereas in our case the disease was confined to lymph nodes. Treatment regimens varied, with most patients receiving lymphoma-directed chemotherapy, though outcomes were generally poor. The addition of this case from Asia broadens the geographic spectrum of the phenomenon and suggests that this association is not restricted to a particular population or ethnicity but may represent a universal pathogenetic interaction. The small number of reported cases limits the ability to draw definitive conclusions about prognosis. However, the uniformly poor outcomes suggest that the coexistence of AITL and KS may reflect particularly severe immune dysregulation and portend an adverse clinical course. Future collection of similar cases will be essential for clarifying whether KS occurrence in the setting of AITL represents merely a coincidental event or whether it defines a distinct clinicopathologic subset with unique prognostic implications. Previous literature documents rare co-occurrences of KS with other lymphoproliferative disorders, including multicentric Castleman disease [21,22,23], Hodgkin lymphoma [24,25], intravascular lymphoma [26], primary effusion lymphoma [27], diffuse large B-cell lymphoma [28,29], and angioimmunoblastic lymphadenopathy [30]. Most involved B-cell lineage neoplasms; among peripheral T-cell lymphomas, AITL is the only subtype reported in association with KS. A comparison of all reported cases, including the present case, is summarized (Table 1). Regarding coexistence of KS and multicentric Castleman disease, Abe et al. [31] described an unusual and instructive case of a 35-year-old HIV-positive man who simultaneously developed KS and multicentric Castleman’s disease (MCD) within the same lymph node. KS, which is typically driven by latent infection of Kaposi’s sarcoma-associated herpesvirus (KSHV), was found to contain spindle cells expressing the latent antigen LANA but almost no lytic proteins. In stark contrast, the MCD lesion—composed of mantle zone B cells with follicular hyperplasia, hyaline vascular changes, and plasma cell proliferation—expressed both latent and multiple lytic KSHV proteins, including ORF50, ORF59, K8, and viral interleukin-6 (vIL-6). Uniquely, vIL-6 was also detected in follicular dendritic cells, likely trapped as immune complexes rather than produced directly. Laboratory analysis confirmed a high KSHV DNA load and elevated human IL-6 levels in the blood, both of which correlated with the patient’s symptoms. Treatment with pegylated-liposomal doxorubicin rapidly resolved the KS lesions, reduced lymphadenopathy from MCD, and sharply lowered both viral load and IL-6 levels. This case not only highlights the contrasting virological states of KSHV in KS (latent) versus MCD (lytic) but also underscores the potential for therapies targeting viral load to benefit both conditions simultaneously.

The diagnosis of synchronous AITL and KS is fraught with challenges. The polymorphous infiltrate of AITL can obscure the spindle cell proliferation of KS, particularly in small biopsy samples. Conversely, the vascular proliferation and red blood cell extravasation of KS may be mistaken for reactive vascular changes in AITL. Immunohistochemistry is indispensable in resolving these diagnostic dilemmas: markers such as PD-1, Bcl-6, and CD10 highlight the TFH nature of AITL [4,5,32], whereas HHV-8 LANA-1, ERG, CD31, and CD34 confirm KS [12]. The use of next-generation sequencing further strengthens the diagnosis by demonstrating canonical mutations such as TET2 and RHOA in AITL [8,10,17]. Failure to recognize the coexistence of two distinct neoplasms can have significant therapeutic consequences. A diagnosis of AITL alone may lead to chemotherapy regimens that are not effective against KS, while a diagnosis of KS alone may lead clinicians to overlook aggressive systemic lymphoma. Thus, awareness of the potential for such collision tumors and the careful application of advanced diagnostic modalities are crucial for accurate classification and management.

Treatment of AITL remains challenging. Standard anthracycline-based regimens such as CHOP are associated with limited efficacy, and novel agents targeting epigenetic regulators, immune checkpoints, and T-cell signaling pathways are under investigation [3]. Autologous stem-cell transplantation may be considered in younger patients achieving remission, but outcomes remain unsatisfactory overall. For KS, treatment depends on the extent of disease and the immune status of the host; options include reduction in immunosuppression, antiviral therapy, and cytotoxic chemotherapy such as liposomal doxorubicin or vinblastine [12]. In the setting of synchronous AITL and KS, therapeutic decisions are particularly complex. Agents directed at AITL may not effectively control KS, and vice versa. Furthermore, some cytotoxic agents may exacerbate immune suppression and thereby worsen KS progression. In our patient, vinblastine was administered, but clinical deterioration continued. Advanced age, frailty, and the coexistence of two aggressive neoplasms contributed to a poor outcome, and palliative care was ultimately the most appropriate course.

This case underscores the importance of individualized therapy in elderly patients with complex hematologic and oncologic diagnoses. Aggressive combination chemotherapy may not be tolerable or effective, and the focus should shift toward symptom control and quality of life. Future research should aim to identify therapeutic strategies that can simultaneously address both the lymphoid malignancy and the virus-driven vascular tumor, perhaps through immune-modulatory or antiviral approaches.

The rarity of collision tumors composed of AITL and KS limits the ability to generalize from individual cases. However, each case contributes valuable insight into the interplay between immune dysregulation and viral oncogenesis. For pathologists, the lesson is to remain vigilant for the possibility of dual pathology in lymph node biopsies, especially when morphological features are heterogeneous. For oncologists, the recognition of such associations underscores the need for comprehensive diagnostic workups that integrate histology, immunohistochemistry, molecular testing, and clinical context. From a broader perspective, this case highlights how intrinsic immune dysregulation within a lymphoma can mimic or substitute for the immunodeficiency typically seen in HIV infection or transplantation. It raises the possibility that AITL may serve as a model for understanding virus-associated oncogenesis in non-HIV settings. Further studies examining the prevalence of latent HHV-8 infection in AITL patients and the potential for viral reactivation could shed light on the mechanisms that link these two malignancies.

## 4. Conclusions

This case represents the first reported occurrence in Asia and the fourth worldwide of a collision tumor comprising AITL and KS in an HIV-negative patient. It reinforces the concept that the immune dysregulation intrinsic to AITL can create a permissive environment for HHV-8 reactivation and KS development. The recognition of this association is critical not only for accurate diagnosis but also for the formulation of realistic therapeutic goals and prognostic expectations. As more cases are reported, a clearer understanding of the clinical and biological significance of this rare phenomenon will emerge, potentially guiding future therapeutic innovations.

## Figures and Tables

**Table 1:** Reported cases of AITL and KS, including present case.

Case	Age/Sex	Ethnicity	Biopsy Site	Skin Lesion (KS)	Clinical Status	HIV Status
1	76/M	Caucasian (Switzerland)	Inguinal LN	-	Stage IV; R-CHOP	Negative
2	49/M	African (Cameroon)	Axillary LN	-	Stage IV; CHOP	Negative
3	70/M	Comorian (Mayotte)	Inguinal LN	+	Stage IV; Palliative care	Negative
This case	81/F	Asian (Korea)	Inguinal LN	-	Stage IV; Palliative care	Negative

## Data Availability

The data presented in this study are contained within the article.

## Abbreviations

LN, lymph node; KS, Kaposi sarcoma; R-CHOP, rituximab + cyclophosphamide, doxorubicin, vincristine, prednisone; CHOP, cyclophosphamide, doxorubicin, vincristine, prednisone.
